# Biosurfactants: Eco-Friendly and Innovative Biocides against Biocorrosion

**DOI:** 10.3390/ijms21062152

**Published:** 2020-03-20

**Authors:** Grażyna Płaza, Varenyam Achal

**Affiliations:** 1 Silesian University of Technology, Faculty of Organization and Management, 26 Roosevelt street, 41-800 Zabrze, Poland; 2 Environmental Engineering Program, Guangdong Technion – Israel Institute of Technology, Shantou 515000, China; varenyam.achal@gtiit.edu.cn

**Keywords:** corrosion, biocorrosion, biosurfactants, biofilm, microbiologically induced corrosion (MIC)

## Abstract

Corrosion influenced by microbes, commonly known as microbiologically induced corrosion (MIC), is associated with biofilm, which has been one of the problems in the industry. The damages of industrial equipment or infrastructures due to corrosion lead to large economic and environmental problems. Synthetic chemical biocides are now commonly used to prevent corrosion, but most of them are not effective against the biofilms, and they are toxic and not degradable. Biocides easily kill corrosive bacteria, which are as the planktonic and sessile population, but they are not effective against biofilm. New antimicrobial and eco-friendly substances are now being developed. Biosurfactants are proved to be one of the best eco-friendly anticorrosion substances to inhibit the biocorrosion process and protect materials against corrosion. Biosurfactants have recently became one of the important products of bioeconomy with multiplying applications, while there is scare knowledge on their using in biocorrosion treatment. In this review, the recent findings on the application of biosurfactants as eco-friendly and innovative biocides against biocorrosion are highlighted.

## 1. Introduction

Corrosion poses a serious hazard on the mechanical structure of buildings, transportation, piping, and automotive parts, among others. The microbiologically induced corrosion (MIC; biocorrosion) caused by both the aerobic and anaerobic bacteria is the most popular in different industrial sectors. Monitoring and control of biocorrosion cost billions of dollars every year. In the last few years, the restrictions on the use of traditional pretreatments and organic coating for corrosion protection have been strictly limited due to both human health and environmental concerns. The threshold values for the dangerous and hazardous substances involved in the production of the most common pretreatments and organic coatings are becoming more stringent year by year. However, most of the traditional chemical inhibitors, such as amines, amides, and quaternary ammonium salts are toxic and harmful. They do not have a favorable environmental profile, and they are able to get bioaccumulated. Most chemicals also lack the required level of biodegradability imposed by legislation. The corrosion inhibitor component is now classified as environmentally friendly according to the following criteria: toxicity, bioaccumulation, and biodegradation. Considering the increasing environmental concerns, the research studies are focused on producing and testing corrosion inhibitors that meet these conditions. The need of alternatives to conventional protection systems promoted a huge number of studies and investigations aiming at the development of innovative and effective solutions with low environmental impact. The known hazardous effects of most synthetic organic inhibitors and the need to develop cheap, nontoxic, and eco-friendly processes have now urged researchers to focus on the use of natural products. There is a need to develop new generation coatings for improved performance and environmental protection. The current strategies using chemical biocides to kill microbes, especially sulphate-reducing bacteria (SRB), have shown great success [1]. However, the use of chemical-based materials imposes hazards on the environment and humans, and current research is focusing on producing green naturally synthetized biocides. This has prompted the search for green corrosion inhibitors as eco-friendly. There are several papers that describe the use of natural resources, ranging from waste materials to plant extracts, as green corrosion inhibitors [1,2,3,4,5,6].

Due to the limitations related with the use of chemical biocides, it is urgent to find new products with antimicrobial properties based on natural sources. This will allow us to replace the risks associated to chemical products. Therefore, the solution for this problem can be solved with the use of natural compounds with antimicrobial properties mainly produced by metabolic mechanism of microorganisms. In fact, it can be an effective alternative solution for the traditional chemical biocides’ substitution. In particular, biosurfactants are biological surface-active compounds, which present environmentally friendly properties, such as low toxicity and high biodegradability.

In this review, the biosurfactants as eco-friendly and innovative biocides against biocorrosion are presented. Some practical examples from the literature, using the biosurfactants as green and smart biocides, are discussed.

## 2. Corrosion Versus Biocorrosion

Corrosion is a global problem that affects a large variety of industries, such as oil refinery, construction and building, sewage, drinking water systems, shipping, etc. Corrosion of materials takes place in the presence of oxygen and moisture, and it is an electrochemical process consisting of an anodic reaction involving the ionization (oxidation) of the metal (the corrosion reaction), and a cathodic reaction based on the reduction of chemical compounds [7]. Finally, it alters the properties of the material and impairs its function [8,9]. Corrosion occurs spontaneously, and it is an old and problematic industrial dilemma, the main cause of the failures of metallic structures [10]. Electrochemical corrosion entails the oxidation and dissolution of a zero valent metal, at a point known as the anode, and a subsequent reduction at the cathode, involving an eternal electron acceptor [9,11].

**Figure 1:**
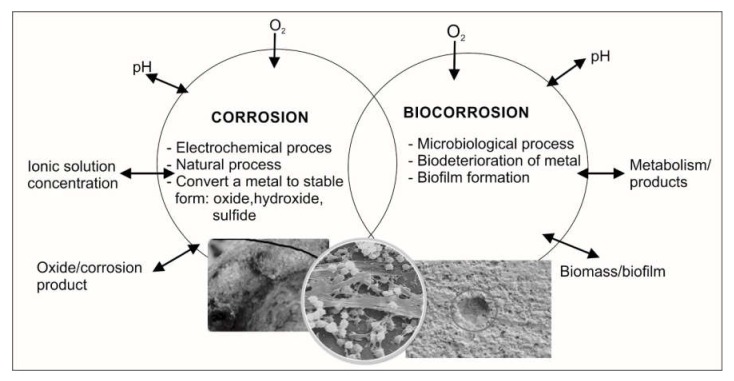
Corrosion versus biocorrosion.

Among the different corrosion mechanisms, microbiologically induced corrosion or microbiologically influenced corrosion (MIC, biocorrosion) is the most popular and closely related to the complex processes connected with the activity of microorganisms (Figure 1). Biocorrosion is a well-established, highly destructive phenomenon. Published cases link bacteria and fungi to accelerated corrosion of steel and east iron, copper alloys, stainless steels, aluminum, and nickel alloys. In addition, microorganisms can cause the destruction of plastics, stone, concrete, wood, etc. They are known to have economic, environmental, and social implications. Microorganisms have the beneficial aspects with relevance to biogenesis of minerals and bioleaching, but many types of microorganisms are also responsible for degradation and corrosion. The microbial corrosion (biocorrosion) processes are the result of electrochemical reactions that are influenced or driven by microorganisms, which are often present as biofilm. Many of the commercially used metals and alloys, such as stainless steels and nickel- and aluminum-based alloys, as well as materials such as concrete, asphalt, and polymers, are readily degraded by microorganisms. Moreover, some protective coatings, oils, and emulsions are subject to microbial degradation.

The destruction microbial processes of inorganic and organic materials can be categorized into biofouling, biodeterioration, and microbiologically influenced corrosion (MIC). Biofouling refers to attachment of micro- and macro-organisms onto material surfaces in marine, freshwater, and soil environments, leading to the formation of fouled layers of biofilms. Biodeterioration is termed as deterioration of nonmetallic materials, like cement, wood, plastics, and rubber, due to microbial action. Microbially influenced corrosion (MIC) takes place in various environments, such as soil, freshwater, and sea water, and it is responsible for more than 30% of all corrosion damage. In this process, the microorganisms stimulate corrosion by consuming the hydrogen and through the secretion of enzymes and acidic metabolites [12]. The main types of bacteria associated with the corrosion are sulfate-reducing bacteria, sulfur–sulfide oxidizing bacteria, iron-oxidizing/reducing bacteria, manganese-oxidizing bacteria, and bacteria producing organic acids, exopolymers, or slime. Microbial-influenced corrosion is always associated with biofilm. These bacteria coexist in naturally occurring biofilms, often forming synergistic consortia with complex interactions. During the biocorrosion, microorganisms, through co-operative metabolism, initiate, facilitate, or exacerbate the corrosion reactions, and they form a biofilm on the surface [13,14,15]. Microbe–metal interactions lead to initial adhesion and biofilm formation. A biofilm can be thought of as a gel composed of 95% water, containing extracellular polymeric substance (EPS) and a suspension of cells and inorganic matter [12]. The five steps of biofilm formation are highlighted as (i) reversible attachment, (ii) irreversible attachment, (iii) beginning maturation, (iv) mature biofilm, and (v) dispersal of planktonic cells. The diversity and growth of microbial community within the biofilm is strongly dependent on environmental factors. The oxygen gradients throughout the biofilm allow growth of both aerobic (at the upper zones) and anaerobic (at lower zones near the substrates) microorganisms. Biofilms modify the properties at the interface between the metal and the bulk solution, such as by changing the types and concentrations of ions and oxygen and pH, leading to a change in the electrochemical behavior of the metal [16]. Microorganisms perform redox reactions that also have a significant effect on the properties of minerals in the environment [17]. The biofilm produced by the microorganisms facilitates biocorrosion by altering various parameters, such as pH, pressure, oxygen levels, and nutrients. In recent years, it has been known that microbes do not only cause corrosion but can also inhibit or protect against corrosion; this process is referred to as microbiologically influenced corrosion inhibition (MICI). Then, the biofilm, as the multi-species combination of microbes, can be used to alter the conditions at a metal surface or produce antibacterial agents, and as a consequence, it can accelerate or inhibit the corrosion process.

In the review of Lin and Ballim [18], the proposed mechanisms on how bacteria contribute to the biocorrosion processes are discussed, and different strategies for biocorrosion control are presented. Moreover, Kip and van Veen [12] described several approaches as potential MIC-inhibition mechanisms. The formation of protective biofilm is mentioned as the first. Biological-control strategies, such as biocompetitve exclusion and the use of antimicrobial-producing biofilm-forming, bacteria show increasing promise as more effective, environmentally friendly, and long-term methods of corrosion controls. The second MICI mechanism is the process of microbially induced precipitation of compounds to protect the material against corrosion. The several examples of naturally formed mineral precipitation layers are described in this paper. MIC has relevance in almost every major industry, with various biological, physical, and chemical strategies used for their control.

**Figure 2:**
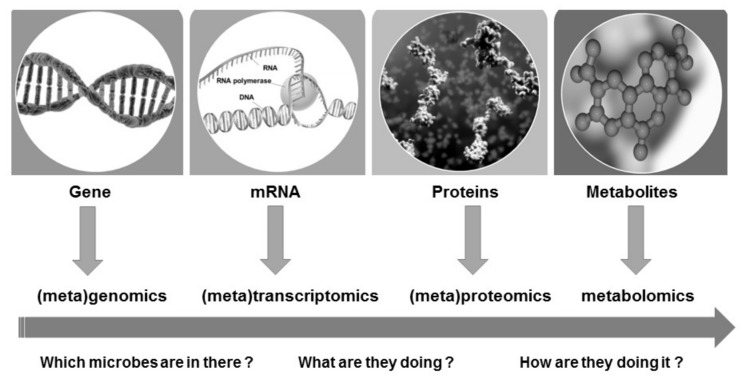
Meta-approaches for investigations of biocorrosion process.

While corrosion itself is a relatively simple process, the investigation of this process in situ is difficult and complicated. Similarly, biocorrosion is influenced by complex processes of microorganisms performing different electrochemical reactions and secreting various secondary metabolites (microbiological/biofilm processes). Up until now, traditional microbiological culture-dependent methods and electrochemical/physical techniques have provided some insights into corrosion activities. However, the identity and role of microbial communities, which are related to corrosion in different materials and in different environments, are scarce. Given the achievements of modern science, it has become possible to gain insight into microbial communities and their metabolism, through the application of omics-based approaches. The review of Beale et al. [19] discusses the recent progress in omics-based applications, to improve the fundamental understanding of biofilms and MIC processes, and it provides a summary of omics-based techniques applied to the MIC investigations. In Figure 2, the omics-based approaches and their role in understanding and characterizing biofilm and MIC are presented. The mechanisms of MIC and MIC inhibition are not completely understood, because they cannot be linked to a single biochemical reactions or specific microbial species or group [12]. The multi-omics methods enable us to study MIC biofilm communities at both their compositional and functional levels. They can be used to characterize and understand MIC biofilms. Till so far, relatively few omics-MIC studies have been reported. The better knowledge of the role of microorganisms in MIC and MIC processes such as biofilm formation and corrosion are required, and that knowledge will be realized through the application of multi-omics research [19].

## 3. Biosurfactants as Green Biocides

Due to limitations related to the use of chemical biocides, it is urgent to find the new products based on natural sources and with appropriate properties like effective antimicrobial activity, economically feasibility, low toxicity, and environmentally friendly features.

Antimicrobial agents produced by microorganisms are among the most powerful bioactive molecules, and their discovery was considered to be one of the greatest achievements of the twentieth century. Since their discovery, a variety of broad- and narrow-spectrum antimicrobial agents have been used worldwide in agriculture, human medicine, and industry, to destroy or inhibit the proliferation of undesirable microorganisms [20,21].

One of the popular antimicrobial agents is surfactants, which are produced synthetically and biologically. Biosurfactants (biologically derived surfactants) are the secondary metabolites and surface-active amphiphilic compounds of biological origin, synthesized by specific bacteria, fungi, and yeasts, with *Pseudomonas aeruginosa*, *Bacillus subtilis*, *Candida albicans*, and *Acinetobacter calcoaceticus* as dominant species [22,23]. The compounds can be secreted into the external environment, form part of the cell membrane, or be metabolized within the cell [24]. They are non-ribosomally synthesized compounds that display noticeable emulsification and surface activities. Biosurfactants form a diverse group of biomolecules with molecular weights ranging from 500 Da to 1000 kDa. Based on their chemical composition and microbial origin, biosurfactants have been classified into different groups. There are five major classes: glycolipids, lipopeptides, phospholipids, polymeric compounds, and neutral lipids [25]. The different chemical compositions of various biosurfactants contribute to their unique physicochemical attributes. Biosurfactants are composed of biological–chemical complexes that include a wide range of molecules, such as fatty and dicarboxylic acids, fatty acid amides, lactones, alkylglycosides, phospholipids, glycolipids, lipopeptides, and sugar molecules. Generally, the molecular components of the biosurfactant are divided into hydrophobic and hydrophilic moieties. The hydrophobic moiety usually consists of saturated or unsaturated long-chain fatty acids, while the hydrophilic moiety is made up of anions, cations, amino acids, or polysaccharides [26,27]. They all show diverse emulsification, interfacial, and surface tension properties. These compounds are known to exhibit broad-spectrum antimicrobial activity, and different classes of biosurfactants are being used by the agricultural, oil, food, cosmetic, biotechnological, and pharmaceutical industries, as well as in a wide range of environmental remediation technologies [24,28,29,30,31,32,33,34,35]. The review of Fenibo et al. [36] offers an overview of diverse applications of biosurfactants, with emphases on petroleum biotechnology, environmental remediation, and the agriculture sector. Once synthesized by the microorganism, the biosurfactants are either secreted extracellularly or are partially attached to the membrane of the cell [37]. The latter arrangement commonly occurs when the microorganism is cultured in water-insoluble substrates. Intracellular biosurfactants are hypothesized to be used for gene and nutrient uptake, to assist host cells in the neutralization of toxic elements by sequestration, to aid in cell differentiation, and, finally, to facilitate the storage of energy and carbon [38]. Biosurfactants reduce surface tension at the phase boundary of a water-insoluble substrate, thus rendering the substrate available for nutrient uptake and metabolism by the producing organism. In addition, biosurfactants enable microorganisms to move along an interface (liquid–liquid, liquid–solid, and liquid–air) more easily. This feature is a result of the reduction of surface tension between the different phases, thus aiding in the motility of organisms in potentially hostile environments [37,38].

Microbially synthesized surfactants (biosurfactants) have advantages over their synthetic counterparts. These include a low toxicity, high selectivity, and specificity of action at extreme pH and temperatures, as well as extensive foaming properties [39]. In the review of Olasanmi and Thring [40], the unique relationship between biosurfactants and environmental sustainability is discussed. The research of surfactants and biosurfactants is a rapidly developing field to their wide applications, like petroleum oil recovery, corrosion inhibition, water and soil pollutions, medical sciences, life sciences, chemistry, etc. In the review of Malik et al. [20], a brief overview of chemical surfactants, such as corrosion inhibitors, is provided. The most important action in corrosion inhibition is the adsorption of the biosurfactant functional group onto the metal surface. The ability of biosurfactants to adsorb is related to their ability to aggregate to form micelles and to form a protective layer at the metal surface. This layer reduces or prevents corrosion of the materials.

Biosurfactants also destroy microbial cells by directly disrupting the integrity of the plasma membrane or cell wall. The magnitude of such damage to the cell boundary makes it difficult for any target organism to develop resistance to the biosurfactant [41,42]. For example, lipopeptides create pores in the cell membrane of the target organism, creating an imbalance in the movement of ions both into and out of the microbial cell that is lethal to the damaged cell [42]. In addition, lipopeptide biosurfactant compounds produced by *Bacillus* species specifically display growth inhibitory and lytic effects against a broad spectrum of microorganisms. These include Gram-negative and Gram-positive bacteria, fungi, and certain viruses [38,43,44,45]. Glycolipid-based biosurfactants such as rhamnolipids primarily produced by *Pseudomonas* species also display algicidal, anti-amoebal, and zoosporicidal properties. These lipid compounds have also been reported to effectively kill various bacteria, as well as fungi and certain viruses [46,47,48,49]. The antibacterial properties and ability to disrupt biofilms by biosurfactants (rhamnolipids and sophorolipids) and sodium dodecyl sulphate (SDS), in combination with selected organic acids, were investigated by Rienzo et al. [44]. In this study, the results suggest that rhamnolipids and sophorolipids may have different mechanisms of action against bacteria. Rhamnolipids inhibit the growth in the exponential phase, suggesting that they may have an influence on the cell division, while the antimicrobial effects of sophorolipids occur between the exponential and stationary phases. Moreover, the authors reported that biofilms formed by *Pseudomonas aeruginosa*, *Escherichia coli*, *Bacillus subtilis*, and *Staphylococcus aureus* on glass coverslips were disrupted with sophorolipids. The results indicated that sophorolipids have a great potential to be used for disruption of biofilms. Earlier studies have also demonstrated that rhamnolipids can disrupt biofilms formed by *Bacillus pumilus*, while surfactin produced by *B. subtilis* has been shown to inhibit biofilm formed by *Salmonella enterica*, *E. coli*, and *Proteus mirabilis* [50,51].

Biosurfactants’ properties of interest also include wetting and penetrating actions, spreading, hydrophilicity and hydrophobicity, emulsification, de-emulsification, detergency, gelling, foaming, flocculation, low critical micellar concentration (CMC) values, microbial growth enhancement, metal sequestration, and antimicrobial actions [23,52,53,54]. Functional properties of biosurfactants are mainly determined by their structure, e.g., location and size of functional groups, in addition to amphiphilic structures. For example, a change in the structure of a cell wall by microorganisms can produce non-ionic or lipopolysaccharides surfactants in their cell wall. There is a growing interest in the investigations of physicochemical and biological properties of biosurfactants because of their various applications [55]. Numerous studies have indicated that polluted environments such as those contaminated with oil, as well as wastewater treatment plants, yield increased numbers and diversity of biosurfactant-producing microorganisms.

## 4. Application of Biosurfactants as Biocides: Examples from the Literature

*Bacillus* species are able to form biofilms and efficiently secrete a wide range of antimicrobial compounds, such as polymyxin B, gramicidin S, and biosurfactants, which belong to the lipopeptides family. They seem to be promising candidates to produce antimicrobials against sulphate-reducing bacteria (SRB). Jayaraman et al. [56] and Zuo et al. [57] reported that *Bacillus* species (naturally or genetically constructed) can produce antimicrobial compounds within the biofilm, resulting in the inhibition of the growth of corrosion-causing SRB and the decrease in corrosion rate of mild steel. Supernatants of the gramicidin S producers, as well as purified gramicidin S, were shown to inhibit the growth of the SRB [57]. The mechanism of action of these antimicrobial substances was shown to involve outer and cytoplasmic membrane disruption. Therefore, the use of bacteria which produce antimicrobial peptides within the biofilm complex to inhibit SRB colonisation within the biofilm is an attractive and promising preventative technique. The successful implementation of this technique would provide saving in practical applications due to the decreased use of high biocide and corrosion-inhibitor concentrations. The role of various types of biosurfactants produced by microorganisms is summarized in Table 1.

In Dagbert et al. [58], the corrosion of stainless steel in the presence of biosurfactant produced by Gram-negative bacteria *Pseudomonas fluorescens* was studied. Stainless steel is frequently used in the maritime field. The biosurfactant solution delayed the corrosion of stainless steels.

Zin et al. [59] studied the influence of surface-active rhamnolipid biocomplex produced by the *Pseudomonas* sp. PS-17 on the corrosion and the repassivation of a freshly cut Al-Cu-Mg aluminum alloy surface. It was established that rhamnolipid biosurfactant complex, consisting of monorhamnolipid, dirhamnolipid, and polysaccharide biopolymer, effectively inhibited the alloy in synthetic acid rainwater. The efficiency of inhibition became stronger with the increase of biosurfactant concentration. However, the inhibition was minor over the critical micelle concentration. Probably the mechanism of corrosion inhibition was related to the adsorption of biosurfactant on the aluminum alloy surface, with the formation of monolayer barrier film. Previously, it was found that both rhamnolipid biocomplex and the supernatant culture from *Pseudomonas* sp. PS-17 inhibited the corrosion of aluminum D16T alloy in distilled water and in 0.1% sodium chloride [60].

Parthipan et al. [61] used glycolipid biosurfactant as an eco-friendly microbial inhibitor (biocide) for the corrosion of carbon steel (API 5LX), which is extensively used in many sectors of the gas and petroleum industry. Carbon steel is the preference for the gas and oil industry because of its high resistance capacity to corrosion. However, the resistance to corrosion is changed in presence of corrosive microbial species such as sulphate reducing bacteria (SRB), acid producers, manganese oxidizing bacteria (MOB), or iron bacteria (IB). As estimated microbial corrosion takes place about 30%–40% of the total corrosion problems in the oil and gas industry. The Authors estimated that biosurfactant produced by *Pseudomonas stutzeri* F01 has the antibacterial properties of corrosive bacterial strains with a low level of concentration.

Astuti et al. [62] described the study to screen biosurfactants that had the potential to be used as an alternative biocide for biofilm associated with biocorrosion, particularly in the oil and gas industry. Eight biosurfactants belonging to glycolipid and rhamnolipid were obtained from indigenous biosurfactant-producing bacteria isolated from an oil reservoir, and their antibiofilm activity against biofilm associated biocorrosion was determined. Through this research, the biosurfactants can be used in the oil industry not only for enhanced oil recovery but also as alternative biocides.

The study of Purwasena et al. [63] showed that biosurfactant produced by indigenous oil reservoir bacteria *Bacillus* sp. is a good candidate for a new anticorrosion agent. A new antimicrobial agent was being developed by using biosurfactant with antibiofilm activity, to combat biocorrosion. The minimum inhibitory concentration (MIC), minimum biofilm inhibitory concentration (MBIC), and minimum biofilm eradication concentration for 50% eradication (MBEC_50_) of biosurfactant against biofilm forming bacteria isolated from oil reservoir were determined, along with their effect on biofilm community structure and ability to inhibit the corrosion rate of carbon steel.

Another area where the biosurfactants and their producers are used is built heritage. It is of vital importance to develop proper remediation actions in conservation treatment based on environmentally innocuous alternatives for microbiologically contaminated historic materials. The environmentally safe and innocuous alternatives to chemical biocides are needed to commonly use during the conservative interventions. The innovative biological method, in the case of historic materials, is called either biocleaning or bioconsolidation [64]. As presented in the literature, bacteria of the genera *Bacillus* are emerging as an alternative due to their capacity to produce biosurfactants with antagonistic activities against many fungal pathogens [65,66,67,68]. Therefore, these selected microorganisms, or their products, are a potential candidate to be used as a safe, natural green biocide for cultural heritage artworks’ safeguard. *Bacillus* species are worth the treatment because they produce a great diversity of secondary metabolites with high biological activity (bioactive compounds). Specifically, they are known to possess antagonistic activities against many fungal pathogens [69]. Some strains of *Bacillus subtilis* and *B. amyloliquefaciens* are known to produce antifungal peptides that belong to antifungal peptides, such as bacilysin and rhizocticin; antifungal lipopeptides (LPPs) such as surfactins, iturins, and fengycins; and antimicrobial polypeptides such as subtilin [69,70]. Silva et al. [67] isolated iturin-producing strains belonging to *B. subtilis*, *B. amyloliquefaciens*, and *B. pumilus* with high antifungal properties that allowed them to select the strains as potential candidates of safe, natural green biocides for biodegraded cultural heritage artefacts. Furthermore, beside having the ability to disrupt biofilm, biosurfactants also possess the ability to prevent biofilm formation [71]. Biofilm prevention is possibly the best strategy to fight biofilms.

## 5. Conclusions

Innovative research studies are needed to replace the chemically synthesized biocides currently used as anticorrosion agents by green solutions that are eco-friendly and do not have negative effects on the environment and human beings. Discovering the new biological routes to reduce the effect of microbial-induced corrosion is of great worth. Researching the better alternatives and nature-friendly solutions is now a big challenge. Despite the presence of numerous studies on the production of new types of green biocides, researchers are still far from reaching the main goals to produce environmentally friendly biocides like biosurfactants. Adequate, on-site technologies based on non-invasive tools should be also developed.

## Figures and Tables

**Table 1:** Biosurfactants derived from *Bacillus* and *Pseudomonas* strains and their role in biocorrosion process.

Strains	Biosurfactants	Role	References
*Bacillus* species	Lipopeptides	Antagonistic effects against a broad spectrum of microorganisms	[38,43]
*Bacillus amyloliquefaciens, Bacillus subtilis*	Peptide, lipopeptides	Antagonistic effects against many fungal pathogens	[69,70]
*Bacillus subtilis*	Surfactin	Inhibit biofilm formed by *Salmonella enterica*, *E. coli*, and *Proteus mirabilis*	[51]
*Pseudomonas* sp. PS-17	Rhamnolipids	Inhibit corrosion of alloy	[59]
*Pseudomonas* spp.	Rhamnolipids	Algicidal, anti-amoebal and zoosporicidal properties	[47,49]
*Pseudomonas fluorescens*	Biosurfactant	Inhibit corrosion of stainless steels	[58]
*Pseudomonas stutzeri* F01	Biosurfactant	Antibacterial properties to corrosive bacterial strains	[61]
